# Non-linear effects of secondary organic aerosol formation and properties in multi-precursor systems

**DOI:** 10.1038/s41467-022-35546-1

**Published:** 2022-12-22

**Authors:** Masayuki Takeuchi, Thomas Berkemeier, Gamze Eris, Nga Lee Ng

**Affiliations:** 1 School of Civil and Environmental Engineering, Georgia Institute of Technology, Atlanta, GA 30332 USA; 2 School of Chemical and Biomolecular Engineering, Georgia Institute of Technology, Atlanta, GA 30332 USA; 3 School of Earth and Atmospheric Sciences, Georgia Institute of Technology, Atlanta, GA 30332 USA; 4 Present Address: Multiphase Chemistry Department, Max Planck Institute for Chemistry, Mainz, 55128 Germany

**Keywords:** Atmospheric chemistry, Atmospheric science, Atmospheric chemistry

## Abstract

Secondary organic aerosol (SOA) contributes significantly to ambient fine particulate matter that affects climate and human health. Monoterpenes represent an important class of biogenic volatile organic compounds (VOCs) and their oxidation by nitrate radicals poses a substantial source of SOA globally. Here, we investigate the formation and properties of SOA from nitrate radical oxidation of two common monoterpenes, α-pinene and limonene. When two monoterpenes are oxidized simultaneously, we observe a ~50% enhancement in the formation of SOA from α-pinene and a ~20% reduction in limonene SOA formation. The change in SOA yields is accompanied by pronounced changes in aerosol chemical composition and volatility. These non-linear effects are not observed in a sequential oxidation experiment. Our results highlight that unlike currently assumed in atmospheric models, the interaction of products formed from individual VOCs should be accounted for to accurately describe SOA formation and its climate and health impacts.

## Abstract (web summary)

Tiny airborne particles play a critical role in two major environmental issues: climate change and air pollution. Our study highlights the important, yet often overlooked non-linear interaction of organic compounds on the formation of such particles.

## Introduction

Secondary organic aerosol (SOA) represents a substantial fraction of fine particulate matter and contributes to uncertainty in climate forcing, adverse human health, and poor air quality^1–3^. Owing to its high abundance and complex nature, tremendous efforts have been put into elucidating the formation chemistry of SOA^2–7^. Laboratory chamber experiments are typically performed to study the oxidation of one precursor volatile organic compound (VOC) at a time in order to obtain fundamental data on the SOA formation and properties specific to the studied VOC. For almost three decades, SOA mass yield^8–10^ remains one of the most widely used metric to simulate SOA formation in ambient environments. Empirical SOA mass yield data are often parametrized as two-product model^8^ or volatility basis set (VBS) model^11^ based on absorptive partitioning theory^9,10^ to predict SOA mass concentration and chemical composition. Results from many studies to date have advanced the fundamental knowledge in the formation, evolution, and fate of SOA, yet the predictive power of models to simulate SOA mass concentration and properties in the ambient air is far from complete^12–15^. One critical assumption implemented in the simulation of SOA mass concentrations is that SOA is formed independently from each individual precursor VOC in multi-precursor systems^16,17^. In other words, oxidation products formed from different precursors are assumed not to chemically interact with one another, even though there are many SOA precursors in the atmosphere being oxidized and forming SOA simultaneously.

The validity of this critical assumption of linear additivity has been investigated in photo-oxidation studies^18,19^, though no consensus seems to have been reached^8,20,21^. The early study by Odum et al.^8^ showed that SOA mass yields from individual VOCs are linearly additive during photo-oxidation of a mixture of two VOCs (i.e., m-xylene and α-pinene; m-xylene and 1,2,4-trimethylbenzene). The same observation was reported for photo-oxidation of an isoprene and α-pinene mixture^22,23^ and for photo-oxidation of mixtures of α-pinene, limonene, and toluene^24^. On the other hand, compared to the sum of the predicted SOA mass concentration based on individual SOA mass yield of each VOC, enhanced SOA formation (20% higher) during photo-oxidation of a VOC mixture containing myrcene was observed^20^. More recently, McFiggans et al.^21^ observed up to 50% reduction in SOA mass yield from α-pinene when it was oxidized together with isoprene in photo-oxidation experiments. The authors attributed this reduced α-pinene SOA mass yield to the lower hydroxyl radical (OH·) concentration available for α-pinene oxidation due to isoprene scavenging OH·. It was also shown that in the presence of isoprene, α-pinene peroxy radicals (RO_2_·) could have dominantly reacted with isoprene RO_2_· rather than with other α-pinene RO_2_·, which hindered the formation of low-volatility α-pinene dimers that would have otherwise contributed to SOA mass concentration. Voliotis et al.^25^ expanded the study to include *o*-cresol and observed an enhanced SOA mass yield for the *o*-cresol−α-pinene system but observed additivity in the *o*-cresol−isoprene system and the *o*-cresol−α-pinene−isoprene system. Taken together, it appears that SOA mass yield in the simultaneous photo-oxidation of multiple VOCs is not always linearly additive, and that such multi-precursor effects can be strongly dependent on precursor identity and oxidation conditions.

Monoterpenes are an important class of biogenic VOCs (BVOCs) and represent one of the dominant SOA precursors worldwide^2,26,27^. In particular, nitrate radical (NO_3_·; formed from reaction of NO_x_ and O_3_) oxidation of monoterpenes is an important and dominant SOA source at night in monoterpene-rich environments^28–30^, representing a direct mechanism linking anthropogenic and biogenic emissions. Globally, the SOA production rate via nitrate radical oxidation of BVOCs is estimated to be over 3 Tg yr^−1^ ^17,31^. Interactions of anthropogenic (e.g., NO_x_) and biogenic emissions (e.g., BVOCs) have been shown to have synergistic effects on SOA formation and have received increased attention as an important source of SOA in the atmosphere^12,28,32–35^. Despite the potential importance, the multi-precursor effects have not been examined for nitrate radical oxidation systems to the best of our knowledge. Elucidating and obtaining a fundamental mechanistic understanding of the chemical interactions of oxidized products in multi-precursor systems is vital to accurately simulate ambient SOA formation and predict its impact on climate and human health.

In this study, we investigate multi-precursor effects in the nitrate radical oxidation of monoterpenes and compare simultaneous and sequential oxidation of the two most common precursor VOCs: α-pinene and limonene. Specifically, we examine mass yield, chemical composition, and volatility of SOA formed in an environmental chamber. The changes in SOA mass yields are analyzed in the context of whether SOA mass yields in multi-precursor systems can be predicted from mass yields of single-precursor systems. Chemical formulae (measured by mass spectrometry) are a common proxy to estimate physicochemical properties of SOA^36,37^ and are, therefore, used to evaluate the difference in chemical composition between single- and multi-precursor systems. The variation in SOA volatility is examined by a comparison of thermograms obtained via thermal desorption coupled with mass spectrometry. Through these comprehensive characterizations, we show that the interaction of oxidation intermediates and products formed from individual VOCs affects the formation and properties of SOA in multi-precursor systems.

## Results

### Analysis of SOA mass yield in multi-precursor systems

**Fig. 1:**
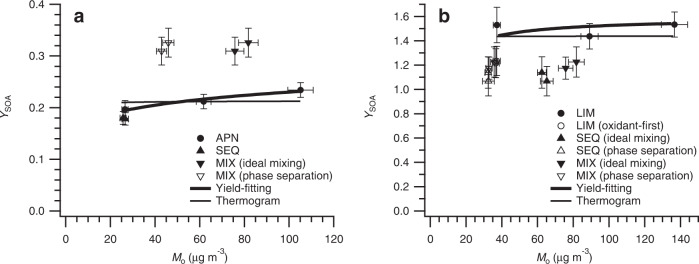
Secondary organic aerosol (SOA) mass yield (*Y*_SOA_) vs. organic mass (*M*_o_). *Y*_SOA_ as a function of *M*_o_ for **a** α-pinene+NO_3_· system from pure α-pinene (APN), sequential oxidation (SEQ), simultaneous oxidation (MIX) experiments and for **b** limonene+NO_3_· system from pure limonene (LIM), SEQ, and MIX experiments. LIM (oxidant-first) refers to the experiment where N_2_O_5_ was first injected into the chamber and followed by the injection of limonene, resembling the limonene oxidation condition in the SEQ experiment. Ideal mixing refers to the assumption of the condition where α-pinene and limonene SOA are ideally mixed (thus *M*_o_ is the total SOA mass concentration), whereas phase separation refers to the assumption of the condition where α-pinene and limonene SOA are phase-separated (thus *M*_o_ is the individual SOA mass concentration). SOA mass yield curves are derived from the volatility basis set (VBS) yield-fitting method (thick solid line; using three data points at varying *M*_o_) as well as the volatility distribution via thermogram analysis combined with the yield data points from APN and LIM experiments (thin solid line). Error bars for *M*_o_ represent the combined uncertainty of a scanning mobility particle sizer (SMPS) and SOA density. Error bars for *Y*_SOA_ are calculated by the standard propagation of error.

SOA mass yield (*Y*_SOA_) is defined as the mass of organics formed (Δ*M*_o_) per mass of precursor VOC reacted (ΔVOC)^8^. It is one of the most fundamental parameters in describing SOA formation and a critical concept is that *Y*_SOA_ is strongly dependent on the organic mass present in the system (*M*_o_) in general^8–10^. Figure 1 shows SOA mass yield data points from nitrate radical oxidation of different monoterpene systems: oxidation of either pure α-pinene or pure limonene (labeled as APN or LIM), sequential oxidation where limonene is oxidized following α-pinene oxidation (SEQ), and simultaneous oxidation of an α-pinene and limonene mixture (MIX). Figure 1a shows the α-pinene SOA mass yield (Δ*M*_o,α-pinene_/Δα-pinene) in the APN, SEQ, and MIX experiments, whereas Fig. 1b shows the limonene SOA mass yield (Δ*M*_o,limonene_/Δlimonene) in the LIM, SEQ, and MIX experiments. Detailed calculation of α-pinene and limonene SOA mass yields in the SEQ and MIX experiments is described in Methods section. In brief, in the SEQ experiment, α-pinene SOA mass yield is obtained in the same manner as in a single-precursor system because α-pinene is oxidized first and forms SOA without limonene. For the rest, differences in aerosol mass spectrometer (AMS) mass spectra of α-pinene and limonene SOA are used to estimate the mass fractions of α-pinene and limonene SOA contributing to the total SOA mass and, thus, SOA mass yield from each precursor VOC.

To facilitate comparison and evaluate whether α-pinene or limonene SOA mass yields in the SEQ and MIX experiments are in line with those in the APN and LIM experiments, it is crucial to know the SOA mass yield curves for pure α-pinene and pure limonene systems because *M*_o_ differs in each experiment. These yield curves (thick solid lines, spanning a wide range of *M*_o_) are also shown in Fig. 1 and are calculated based on three experiments with differing initial VOC concentrations (APN-1, APN-2, APN-3 for the α-pinene SOA mass yield curve and LIM-1, LIM-2, and LIM-3 for the limonene SOA mass yield curve; Supplementary Table 1). The VBS coefficients representing these yield curves are shown in Supplementary Table 2. The yield curves are independently evaluated by a comparison with those derived from the volatility distribution of oxidation products estimated via thermogram analysis, using the yield data points from the APN and LIM experiments as constraints (see Methods section). Any substantial deviation of SOA mass yield data points in the SEQ and MIX experiments from either yield curve can therefore be attributed to multi-precursor effects, i.e., the change in SOA mass yield that cannot be accounted for by simply using SOA mass yields obtained at respective *M*_o_ in single-precursor systems.

The SOA mass yield of α-pinene in the SEQ experiments is in agreement with the yield curves (Fig. 1a). Note that α-pinene is first oxidized before limonene addition in the SEQ experiment. Thus, it is expected that α-pinene SOA mass yield from the SEQ experiment agrees with the yield curves. α-pinene SOA mass yield in the MIX experiment shows an enhancement (~50%) compared to the yield curves, indicating changes in the α-pinene SOA formation pathways in the presence of limonene. Reduced limonene SOA mass yields are observed during both sequential oxidation (SEQ) and simultaneous oxidation (MIX) of α-pinene and limonene by nitrate radicals (Fig. 1b). In the SEQ experiment, the injection order of VOC and oxidant is different from the LIM experiments; limonene is added to the chamber with leftover N_2_O_5_ from α-pinene oxidation. It has been reported that the injection order of VOC and oxidant affects the SOA formation chemistry and thus the SOA mass yield for nitrate radical oxidation of isoprene, owing to different RO_2_· fates^31^. Additional experiments (LIM-4 and LIM-5; Table 1 and Supplementary Table 1), where N_2_O_5_ is first injected followed by limonene injection, show the same limonene SOA mass yield as the SEQ experiment. Therefore, the lower limonene SOA mass yield in the SEQ experiment than the yield curve is likely attributed not to the presence of α-pinene but to the injection order of VOC and oxidant. In the MIX experiment, limonene SOA mass yield is lower than the predicted yield curves. The measured limonene SOA mass yield in the MIX experiment is 1.18–1.23 (at *M*_o_ = 76–82 µg m^−3^), whereas the predicted yield at same *M*_o_ is 1.51. This suggests that certain limonene SOA formation pathways are hindered in the presence of α-pinene.

**Table 1:** Summary of experimental conditions

Expt.	Experiment variant	α-pinene (µg m^−3^)	limonene (µg m^−3^)	N_2_O_5_ (ppb)	NO_2_ (ppb)
APN-1	Pure α-pinene, low conc.	135.1 ± 9.5	—	100	83.5
APN-2	Pure α-pinene, med conc.	291.4 ± 11.1	—	200	154.9
APN-3	Pure α-pinene, high conc.	449.4 ± 13.5	—	300	219.6
LIM-1	Pure limonene, low conc.	—	24.3 ± 1.9	40	30.0
LIM-2	Pure limonene, med conc.	—	62.0 ± 3.0	80	54.9
LIM-3	Pure limonene, high conc.	—	89.1 ± 3.6	120	78.9
LIM-4	Pure limonene, low conc., oxidant-first	—	30.1 ± 2.5	40	—
LIM-5	Pure limonene, low conc., oxidant-first	—	30.1 ± 2.5	40	—
SEQ-1	Sequential oxidation	142.6 ± 6.1	28.4 ± 2.8	140	110.1
SEQ-2	Sequential oxidation	149.4 ± 6.1	32.6 ± 3.3	140	112.0
MIX-1	Simultaneous oxidation	141.1 ± 9.5	29.2 ± 2.5	140	105.0
MIX-2	Simultaneous oxidation	138.6 ± 9.5	27.9 ± 1.5	140	116.7

Experiment names are based on the type of volatile organic compounds (VOCs) or order of injection. α-pinene and limonene are the initial concentrations measured by a gas chromatograph-flame ionization detector (GC-FID). N_2_O_5_ concentration corresponds to the total amount injected. NO_2_ is the concentration measured after the complete consumption of α-pinene and/or limonene.

Reduction or enhancement in the formation of SOA in multi-precursor photo-oxidation systems has been examined previously with respect to the mixing state of different organic compounds^23,38,39^; however, nitrate radical oxidation systems have not been investigated to date. Here, in the MIX experiment, we observe the same extent of increase of α-pinene and reduction of limonene SOA mass yields, regardless of whether α-pinene and limonene SOA are assumed to be well-mixed or phase-separated (Fig. 1). Therefore, while we cannot pinpoint whether α-pinene and limonene SOA are well-mixed or phase-separated in the SEQ and MIX experiments in this study, these analyses indicate that unlike in previous studies, the mixing state of α-pinene and limonene SOA cannot explain the observed enhancement and reduction in α-pinene and limonene SOA mass yields, respectively. Changes in oxidation condition and radical chemistry have been proposed as another potential reason for reduced SOA mass formation of multi-precursor photo-oxidation systems^21,40,41^. In this study, since the ratio of N_2_O_5_ to initially available double bonds in VOCs is kept constant for all experiments, we do not expect differences in the concentration of nitrate radicals to cause changes in SOA mass yields. Also, RO_2_· fate is roughly the same across all experiments, where reactions with other RO_2_· and nitrate radicals dominate the fate of RO_2_· by similar magnitudes (Supplementary Table 3). Therefore, the altered individual SOA mass yields in the MIX experiment likely do not arise from principal changes in RO_2_· fate. In the following sections, we investigate potential reasons for the enhancement and reduction in α-pinene and limonene SOA mass yields, respectively, in the MIX experiment by comprehensively examining SOA chemical composition and volatility in single- and multi-precursor systems.

### Analysis of SOA chemical composition

**Fig. 2:**
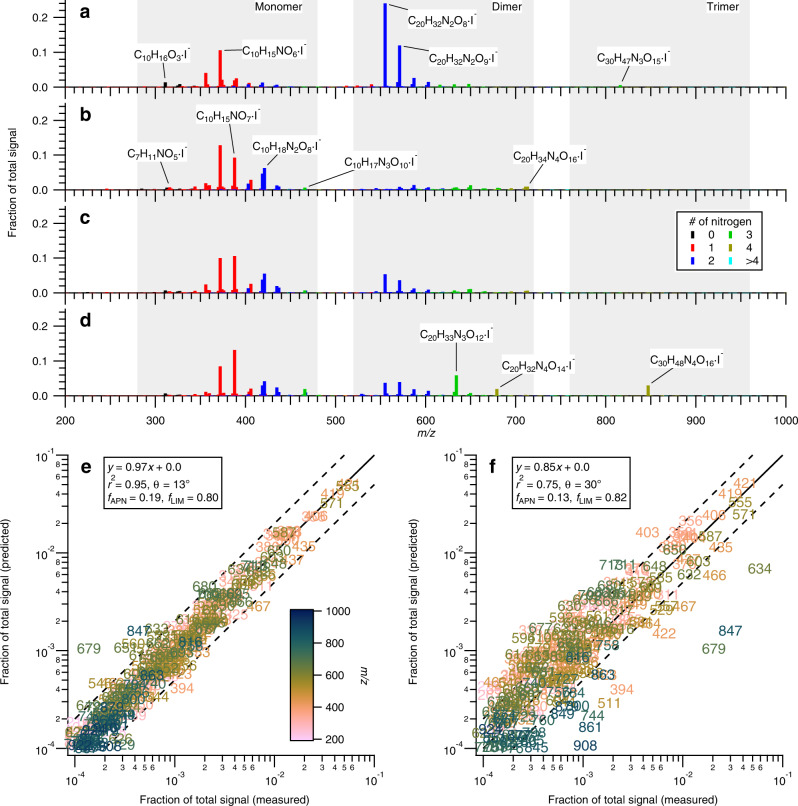
Mass spectra of a filter inlet for gases and aerosols coupled to a chemical ionization mass spectrometer (FIGAERO-CIMS) and their comparison between single- and multi-precursor systems. FIGAERO-CIMS mass spectra in **a** pure α-pinene (APN), **b** pure limonene (LIM), **c** sequential oxidation (SEQ), and **d** simultaneous oxidation (MIX) experiments, colored by the number of nitrogen atoms in the assigned molecular formulae. Monomer, dimer, and trimer regions are shaded. Comparison of the mass spectra observed in **e** SEQ and **f** MIX experiments with those predicted based on linear combinations of APN and LIM experiments. Solid line indicates 1:1 and dashed lines represent a deviation by a factor of 2. Data points are labeled and colored by nominal *m/z*. *θ* is a spectral contrast angle, and *f*_APN_ and *f*_LIM_ indicate the signal fractions of APN and LIM experiments contributing to secondary organic aerosol estimated via multiple linear regression analysis, respectively.

Over the course of each experiment, SOA composition is continuously characterized by a filter inlet for gases and aerosols coupled to a chemical ionization mass spectrometer (FIGAERO-CIMS). Illustrated in Fig. 2a–d and in Supplementary Fig. 1 are FIGAERO-CIMS mass spectra from the APN, LIM, SEQ, and MIX experiments, respectively. Detected species form characteristic monomer, dimer, and trimer compounds, indicating that oligomerization is prevalent during nitrate radical oxidation of both monoterpenes, consistent with prior studies^42,43^. More signals that can be associated with dimer and trimer compounds are detected in the APN experiment (Fig. 2a) compared to the LIM experiment (Fig. 2b). It is important to note that this observation does not necessarily indicate that oligomers are generally more abundant in the APN experiment, but that there is a higher contribution of thermally stable C_20_ and C_30_ accretion products. Less thermally stable oligomers in the LIM experiment may have decomposed due to the thermal desorption technique employed by FIGAERO-CIMS. The detected SOA products from the α-pinene+NO_3_· system are consistent with previous studies^44–47^. A FIGAERO-CIMS mass spectrum of the limonene+NO_3_· system is also available in the literature^48^ and is similar to our study, though our assignments of some chemical formulae at *m/z* > 600 are different. The assignments of chemical formulae beyond the range of mass calibrants (*m/z* > 500) are detailed in Supplementary Information. Below, we first evaluate the molecular-level chemical speciation of SOA in the APN and LIM experiments independently, then proceed to investigate the linear additivity in SOA chemical composition in the SEQ and MIX experiments.

While many compounds with the same chemical formulae (e.g., C_10_H_15_NO_5–7_·I^−^) are detected in both APN and LIM experiments, a number of detected ions are substantially more abundant in either APN or LIM experiment. The major and characteristic ions (ions that are relatively more abundant in each variant of experiment compared to others) detected in the APN experiment are C_10_H_16_O_3_·I^−^, C_10_H_15_NO_6_·I^−^, C_20_H_32_N_2_O_8-9_·I^−^, and C_30_H_47_N_3_O_15_·I^−^. C_10_H_16_O_3_ likely corresponds to pinonic acid, whereas C_10_H_15_NO_6_ is keto peroxyacyl nitrate. These compounds can be formed via oxidation of pinonaldehyde by nitrate radicals, followed by reaction of the resulting RO_2_· with other RO_2_· and NO_2_, respectively^49^. The formation of C_20_H_32_N_2_O_8-9_ is possible through gas-phase RO_2_· + RO_2_· reaction or particle-phase oligomerization of stable products, similar to those proposed for the oxidation of isoprene and β-pinene by nitrate radicals^31,50^. C_30_H_47_N_3_O_15_ is a characteristic compound among trimers in the APN experiment though its relative abundance compared to monomers and dimers is low. Its formation occurs rapidly upon oxidation of α-pinene, concurrent with the formation of some monomers and dimers (Supplementary Fig. 2). Therefore, this C_30_ compound may be formed via gas-phase RO_2_·(C_10_) + RO_2_·(C_20_) reaction or rapid particle-phase oligomerization, such as hemiacetal and peroxyhemiacetal formation^7,51^.

SOA formed in the LIM experiment generally contains a greater number of nitrogen atoms per carbon number (signal-weighted average formula: C_12.2_H_19.0_N_1.6_O_8.2_, N:C = 0.13) than those formed in the APN experiment (signal-weighted average formula: C_15.6_H_24.4_N_1.6_O_7.8_, N:C = 0.10) (Fig. 2), due to the presence of an additional double bond in limonene that can be oxidized by nitrate radicals. Characteristic peaks in the mass spectrum of the LIM experiment correspond to C_7_H_11_NO_5_·I^−^ (hydroxyl carbonyl nitrate), C_10_H_18_N_2_O_8_·I^−^ (di-hydroxyl di-nitrate), C_10_H_17_N_3_O_10_·I^−^ (hydroxyl tri-nitrate), and C_20_H_34_N_4_O_16_·I^−^. C_7_ compounds like C_7_H_11_NO_5_ are likely formed from alkoxy radical decomposition following the nitrate radical addition to the exocyclic double bond. C_10_ di-nitrate compounds are likely generated by nitrate radical addition to both double bonds, and C_20_H_34_N_4_O_16_ can be formed via dimerization reaction of such C_10_ di-nitrate compounds. The production of C_10_ tri-nitrate compounds is surprising given that limonene only bears two double bonds for nitrate radicals to attack. It may be possible for RO_2_· + NO reaction to take place to a small extent. Alkyl radical β-scission of a C-C bond in the six-membered ring would create a third double bond that could be oxidized to introduce another nitrate functional group. Aside from the characteristic ions in the APN and LIM experiments, the relative abundance of the common species with the same chemical formulae also differs greatly between APN and LIM experiments.

The distinct SOA mass spectral features in the APN and LIM experiments enable us to evaluate the linear additivity in SOA chemical composition in the SEQ and MIX experiments. Shown in Fig. 2e is a comparison of the measured SOA mass spectrum in the SEQ experiment with the prediction based on linear combinations of SOA mass spectra from the APN and LIM experiments. The majority of compounds fall on the 1:1 line with some scatter, yet within a deviation of a factor of 2, indicating that the composition of SOA formed in the SEQ experiment can be expressed as linear additions of SOA formed in the pure systems (*r*^2^ = 0.95). Shown in Supplementary Fig. 3 are the time series data of particle-phase species in the SEQ experiment. As expected, the signals for the α-pinene oxidation products increase at the first data point (*t* = 40 min), whereas those of limonene are not detected. Upon introduction of limonene at 60 min, limonene oxidation products start forming and partitioning into the particle phase (*t* = 100 min). The same linear additivity, however, is not observed in the MIX experiment (Fig. 2f). The degree of scatter is evidently greater, with fewer *m/z* on the 1:1 line (*r*^2^ = 0.75). The predicted mass spectrum based on linear combinations of the APN and LIM experiments under-predicts dimeric (*m/z* 520–720, colored in light green) and some trimeric species (*m/z* 760–960, colored in dark green), whereas monomeric compounds (*m/z* 280–480, colored in pink) are slightly over-predicted. Again, a larger prevalence of signals from dimer and trimer compounds in the MIX experiment, compared with the combination of APN and LIM experiments, does not necessarily mean that oligomers are more abundant, but that there is a higher contribution of thermally stable C_20_ and C_30_ accretion products.

These observations point to additional formation pathways of the thermally stable C_20_ and C_30_ accretion products that show enhanced presence in the MIX experiment. We speculate that either (1) cross-reactions of gas-phase RO_2_· formed from α-pinene and limonene are more efficient in generating thermally stable C_20_ and C_30_ accretion products and/or that (2) particle-phase oligomerization reactions involving both α-pinene and limonene oxidation products generate more thermally stable C_20_ and C_30_ accretion products. While the latter could also be possible in the SEQ experiment, particle-phase cross-reactions may be inhibited due to spatial separation of α-pinene and limonene oxidation products (due to a viscous phase state) or if particle-phase chemistry of α-pinene oxidation products has already occurred before limonene chemistry starts.

**Fig. 3:**
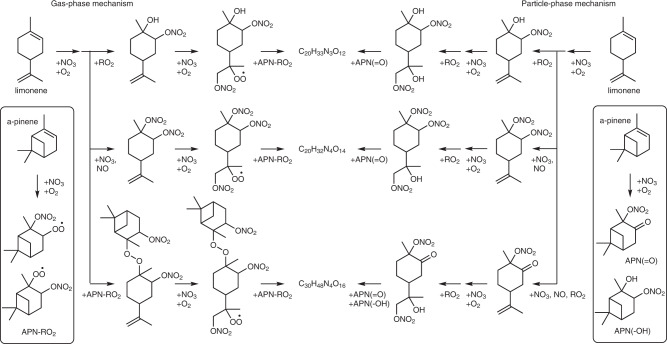
Proposed mechanisms for the formation of C_20_H_33_N_3_O_12_, C_20_H_32_N_4_O_14_, and C_30_H_48_N_4_O_16_ in the simultaneous oxidation (MIX) experiment. Gas-phase mechanism via cross-reactions of α-pinene and limonene peroxy radicals (RO_2_·), and particle-phase mechanism via hemiacetal formation involving α-pinene and limonene oxidation products. APN-RO_2_, APN(=O), and APN(-OH) represent α-pinene oxidation intermediate (i.e., RO_2_·), α-pinene oxidation products with carbonyls, and α-pinene oxidation products with hydroxyl functional groups, respectively.

Three species at *m/z* 634 (C_20_H_33_N_3_O_12_·I^−^), 679 (C_20_H_32_N_4_O_14_·I^−^), and 847 (C_30_H_48_N_4_O_16_·I^−^) show substantially higher abundance in the MIX experiment than in the APN, LIM, and SEQ experiments (Fig. 2a–d). These low-order oligomeric species (dimers and trimers) can be formed via cross-reactions of α-pinene and limonene oxidation intermediates/products in the gas phase and/or in the particle phase (Fig. 3). They are unlikely formed efficiently from the self- or cross-reactions of either α-pinene or limonene oxidation intermediates/products alone, evidenced by much lower abundance of these compounds in the APN and LIM experiments (Fig. 2a, b). Counting double bond equivalents (DBE; where we consider a nitrate functional group to be equivalent to H) in these three oligomeric oxidation products supports the hypothesis that they are hetero-oligomers. The initial number of rings present in α-pinene and limonene are 2 and 1, respectively. Considering that all the initially available double bonds in α-pinene and limonene are to be oxidized by nitrate radicals and that initially present carbon rings remain intact, DBE of resulting α-pinene and limonene oxidation intermediates/products are 2 and 1, respectively. Therefore, DBE of α-pinene and limonene homo-oligomers should be 2*n* and *n*, respectively, where *n* is the number of monomeric units, provided that oligomerization processes neither increase nor decrease DBE. Characteristic compounds in the APN and LIM experiments follow this trend; DBE of C_20_H_32_N_2_O_8-9_ and C_30_H_47_N_3_O_15_ in the APN experiment are 4 and 6, respectively, and that of C_20_H_34_N_4_O_16_ in the LIM experiment is 2. On the other hand, DBE of C_20_H_33_N_3_O_12_, C_20_H_32_N_4_O_14_, and C_30_H_48_N_4_O_16_ are 3, 3, and 5, respectively, and deviate from the expected values for α-pinene and limonene homo-oligomers (4, 4, and 6 for α-pinene homo-oligomers; 2, 2, and 3 for limonene homo-oligomers).

It has been shown previously that the cross-reaction rate of gaseous RO_2_· formed from isoprene and α-pinene oxidation is on the same order of magnitude with that of α-pinene RO_2_·, while the self-reaction rate of isoprene RO_2_· is several orders of magnitude lower^52^. Despite the different combination of precursor VOCs in this study, one could expect a similar enhanced reaction rate between α-pinene and limonene products. The deviation of FIGAERO-CIMS mass spectrum in the MIX experiment from linear combinations of the APN and LIM experiments points towards the role of differing gas-phase and/or particle-phase chemistry when multiple precursor VOCs are oxidized simultaneously.

We conduct a similar comparison analysis using mass spectra data from AMS, which is a widely used instrument to analyze bulk SOA chemical composition in the field of aerosol chemistry. Unlike FIGAERO-CIMS, AMS provides much less detailed molecular-level information owing to prevalent fragmentation by electron impact ionization, which is evident by the much lower *m/z* of detected compounds as well as a smaller range of *m/z* (Supplementary Fig. 4a–d). The AMS mass spectrum of SOA from the pure α-pinene system has characteristic large signals at *m/z* 43 (C_2_H_3_O^+^), *m/z* 83 (C_5_H_7_O^+^), and *m/z* 91 (C_7_H_7_^+^)^44^. On the other hand, AMS mass spectrum of limonene SOA is dominated by *m/z* 43 (C_2_H_3_O^+^)^53^, which is expected to come from the fragmentation of non-acid oxygenates. In contrast to FIGAERO-CIMS data (Fig. 2e, f), the prediction based on linear combinations of AMS mass spectra in APN and LIM experiments shows a good agreement with measured AMS mass spectra in both the SEQ and MIX experiments, indicated by excellent *r*^2^ and a slope that is very close to 1 (Supplementary Fig. 4e, f). This suggests that the bulk organics data by AMS alone are not sufficient to determine whether a multi-precursor system can be expressed as linear combinations of the individual systems for the types of SOA examined in this study. The different responses by FIGAEOR-CIMS and AMS to the linearity analysis could be mainly driven by the type of formed oligomers (e.g., LIM-LIM vs. LIM-APN). Owing to hard ionization leading to prevalent fragmentation, AMS may show very similar mass spectra regardless of the structure of formed oligomers as long as the structures of the monomer blocks are the same. Nonetheless, our analysis of SOA mass composition highlights the importance of complimentary molecular-level, speciated chemical characterization measurements in understanding aerosol chemistry.

### Analysis of thermal desorption profiles and SOA volatility

**Fig. 4:**
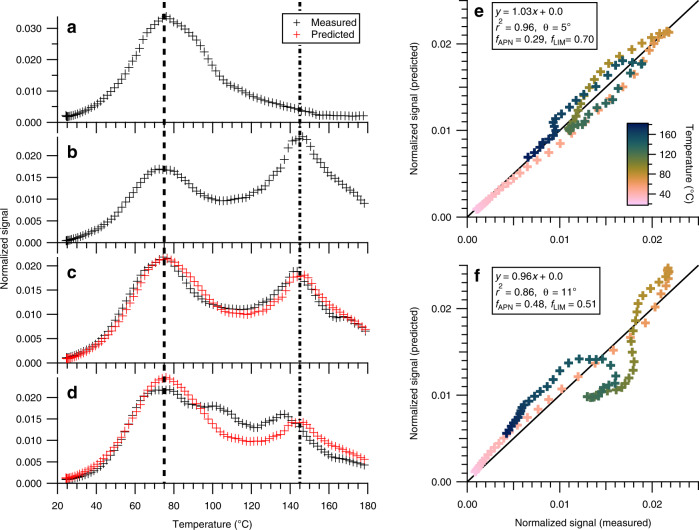
Thermograms of a filter inlet for gases and aerosols coupled to a chemical ionization mass spectrometer (FIGAERO-CIMS) and their comparison between single- and multi-precursor systems. FIGAERO-CIMS thermograms obtained in **a** pure α-pinene (APN), **b** pure limonene (LIM), **c** sequential oxidation (SEQ), and **d** simultaneous oxidation (MIX) experiments. Two dashed lines are shown to visually guide the positions of peak desorption temperature in APN (75 °C) and LIM (145 °C) experiments, respectively. Comparison of measured thermograms in **e** SEQ and **f** MIX experiments with those predicted based on linear combinations of APN and LIM experiments. Solid line represents 1:1. Data points are colored by temperature. *θ* is a spectral contrast angle, and *f*_APN_ and *f*_LIM_ indicate the signal fractions of APN and LIM experiments contributing to secondary organic aerosol estimated via multiple linear regression analysis, respectively.

The thermal desorption technique employed by FIGAERO-CIMS provides volatility information of bulk and speciated SOA because the measured desorption temperatures correlate to vapor pressures^54–56^. Figure 4a–d shows the thermal desorption profiles (also referred to as thermograms) of SOA formed in the APN, LIM, SEQ, and MIX experiments, respectively. To the best of our knowledge, a FIGAERO-CIMS thermogram of SOA formed from nitrate radical oxidation of α-pinene has not been reported in the literature. In this study, we observe a distinct peak at 75 °C (Fig. 4a). The thermal desorption profiles of individual limonene SOA species show two distinct peaks (Fig. 4b) and are consistent with a previous study^48^. The first peak at a lower desorption temperature appears broader than the second peak, resulting from high variability in the volatility of products. The second peak is narrower and has a maximum of ~145 °C. Taken together, these observations indicate that on average, SOA formed in the APN experiment is more volatile than that formed in the LIM experiment. This also suggests that FIGAERO-CIMS mass spectra alone cannot be used to directly quantify the abundance of oligomers, as the mass spectrum of the APN experiment indicates a higher abundance of oligomers in mass spectra than that of the LIM experiment.

SOA in the SEQ experiment shows a thermal desorption profile similar to the combination of SOA in the APN and LIM experiments, with two distinct peaks at desorption temperatures of 75 and 145 °C (Fig. 4c). Indeed, the measured thermogram in the SEQ experiment can be predicted well solely by linear combinations of APN and LIM experiments (Fig. 4e; *r*^2^ = 0.96), similar to the mass spectrum analysis presented in Fig. 2e. The thermal desorption profile of SOA in the MIX experiment, however, shows a different pattern than those in the APN and LIM experiments. The first peak appears at the same desorption temperature but there is an additional peak at 105 °C, which is not present in the APN nor LIM experiment. The third peak is located at a lower desorption temperature (130 °C instead of 145 °C) than the second peak in the LIM experiments (Fig. 4d). As shown in Fig. 4f, the measured thermal desorption profile in the MIX experiment does not match the predicted thermogram based on linear combinations of the APN and LIM experiments (*r*^2^ = 0.86). The signal contributions at 75 °C and 145 °C are predicted to be higher than the measurement, whereas the contribution at 80–130 °C is under-predicted. Thus, similar to the mass spectra analysis, the thermogram analysis suggests that the formation of oxidation products from the pure α-pinene and limonene systems is reduced and that a new group of compounds with different volatility than pure α-pinene or limonene oxidation products are formed in the MIX experiment.

**Fig. 5:**
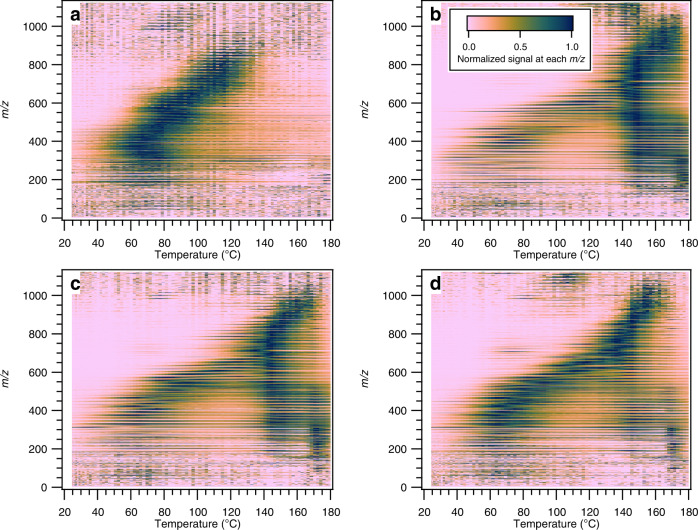
2-D thermograms. Thermograms of a filter inlet for gases and aerosols coupled to a chemical ionization mass spectrometer (FIGAERO-CIMS) at each nominal *m/z* in a 2-D space for **a** pure α-pinene (APN), **b** pure limonene (LIM), **c** sequential oxidation (SEQ), and **d** simultaneous oxidation (MIX) experiments. Signals at each nominal *m/z* are normalized such that the largest signal is 1.

2-D thermograms, plotted as *m/z* vs. desorption temperature and colored with signal intensity, are an effective means to illustrate the shape of thermograms of individual compounds rather than that of bulk SOA^57^. The large signal peak at 75 °C in the APN experiment is mainly from compounds with *m/z* < 600 (Fig. 5a). In the LIM and SEQ experiments, there is a band of high signal intensity at 145 °C spanning from *m/z* < 300 to 900 (Fig. 5b, c). The ubiquitous presence of this high signal intensity band at 145 °C across a wide range of *m/z* suggests that it likely represents the decomposition of high molecular weight, low-volatility oligomers^57,58^. As the desorbing gas temperature increases, these low-volatility oligomers (C_>30_) become unstable and break apart into smaller molecules before they evaporate. Interestingly, there is a lack of this high signal intensity band at 145 °C in the MIX experiment (Fig. 5d). As an example, the absence of the 145 °C peaks for dominant oligomeric compounds in the MIX experiment is illustrated in Supplementary Fig. 5, while the peaks can be observed in the LIM and SEQ experiments. The inhibited formation of low-volatility compounds, likely high-order oligomers (C_>30_), from limonene during simultaneous oxidation can explain the observed reduction in limonene SOA mass yield in the MIX experiment.

**Fig. 6:**
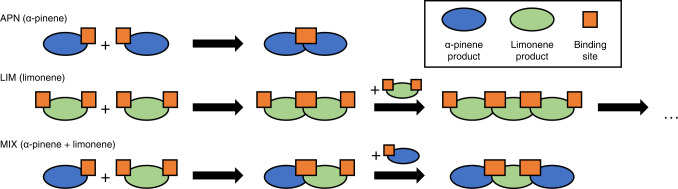
Schematic of oligomerization processes in single- and multi-precursor systems. Limonene oxidation intermediates/products (green) are likely to have twice as many oligomerization sites (orange; i.e., peroxy radicals (RO_2_·) and functional groups) as α-pinene oxidation intermediates/products (blue) due to the higher number of double bonds in the precursor molecule. This may lead to much higher oligomerization degrees for secondary organic aerosol (SOA) formed in pure limonene (LIM) experiment compared to in pure α-pinene (APN) and simultaneous oxidation (MIX) experiments.

A potential explanation for the lack of low-volatility, high-order oligomers in the MIX experiment is that cross-reactions of α-pinene and limonene oxidation products inhibit the formation of larger oligomer chains, and we postulate a simplified mechanism (Fig. 6). The formation of oligomers requires binding sites for the interaction of two molecules. The presence of two double bonds in limonene allows the formation of binding sites at opposite ends of the molecule, while for α-pinene oxidation products this can happen at only one reaction site. Thus, the greater number of double bonds in limonene allows more possibilities for monomeric limonene oxidation products to oligomerize (Fig. 6), or simply a larger chance of oxidation products bearing certain functional groups with a high tendency for oligomerization. In the gas phase, these binding sites are likely RO_2_∙, while in particle-phase oligomerization reactions the binding sites are functional groups such as hydroxyl, hydroperoxide, and carbonyl groups (Fig. 3). Applying this logic, the greater number of functional groups in limonene oxidation products allows the chain of oligomerization to form high-order oligomers (C_>30_), while α-pinene oxidation products, with fewer binding sites, may at most form dimers. This is supported by the findings of our previous kinetic modeling analysis^42^, which inferred a high oligomer content of limonene SOA and a low oligomer content of α-pinene SOA based on thermal evaporation rates. That study also suggested a mechanism in which higher-volatility oxidation products (C* = 1000 µg m^−3^) contributed to limonene SOA mass through a slow process of equilibrium partitioning and subsequent oligomerization, thus explaining the exceptionally high SOA mass yield of limonene with a high propensity of oligomer formation that pulls higher-volatility oxidation products into the particle phase. For the MIX experiment, we propose that the presence of α-pinene oxidation products could obstruct the chain of oligomerization from limonene oxidation products, hence decreasing the limonene SOA yield in the mixture. In turn, the presence of limonene oxidation products can offer additional binding sites for α-pinene oxidation products, hence increasing the α-pinene SOA yield. Taken together with the mass spectral analysis, this demonstrates that the volatility of bulk SOA formed from the simultaneous oxidation of multiple precursor VOCs should not be treated as linear additions of SOA from individual VOCs. Depending on the volatility of resulting oxidation products, SOA mass yield can be greatly enhanced or reduced, as demonstrated in Fig. 1.

## Discussion

In this study, we conduct the first investigation on the linear additivity of SOA formation and properties in nitrate radical oxidation of the two most common monoterpenes: α-pinene and limonene. Specifically, changes in SOA mass yield, chemical composition, and volatility from simultaneous (MIX) and sequential (SEQ) oxidation are examined. Sequential oxidation does not appear to affect SOA mass yield of α-pinene nor limonene. This is evident by the fact that the SOA chemical composition and volatility in the SEQ experiment can be predicted well, solely based on linear combinations of the APN and LIM experiments. On the other hand, α-pinene SOA mass yield is observed to enhance by ~50%, while limonene SOA mass yield is observed to reduce by ~20% in the MIX experiment. Mass spectral and thermogram analyses demonstrate that the formation of low-volatility, high-order oligomers (C_>30_) from limonene oxidation products is inhibited in the MIX experiment, which could explain the reduction in limonene SOA mass yield. Cross-reactions of α-pinene and limonene oxidation intermediates or products in the MIX experiment may effectively terminate the oligomerization chain reaction of limonene oxidation products. On the other hand, the presence of limonene oxidation products can offer additional binding sites for α-pinene oxidation products to promote the formation of hetero-oligomers to enhance α-pinene SOA mass yield. These non-linear interactions during the oxidation of precursors are important even for similar molecules from the same compound group (i.e., cyclic monoterpenes). The current treatment of oxidation of individual VOCs independently contributing to SOA in atmospheric models does not capture and account for the interaction of intermediates and products formed from oxidation of various VOCs. It is crucial that the new model development effort considers the non-linear effects of multi-precursor systems to accurately predict SOA formation in the atmosphere and their climate and health impacts.

In addition, using linear combinations of mass spectra, we show that the molecular-level details provided by FIGAERO-CIMS clearly elucidate changes in SOA chemical composition between single- and multi-precursor experiments, whereas the same analyses with AMS mass spectra yields no evidence for differing SOA composition in the SEQ and MIX experiments. This is potentially due to non-linearity in the SOA formation being driven by the efficient formation of hetero-oligomers. If the monomer blocks are the same regardless of the structure of formed oligomers, the molecular fragments and hence the resulting AMS mass spectra may be similar. Overall, the combination of bulk (AMS) and molecular-level (FIGAERO-CIMS) information is an effective approach to obtain detailed insights into the chemistry of SOA.

SOA mass yield is not a scalar value, but rather strongly depends on the organic mass present in the system (*M*_o_)^8–10^. Emanuelsson et al.^24^ reported that the SOA formation during photo-oxidation of the mixtures of α-pinene, limonene, and toluene could be described as linear combinations of the corresponding single-precursor systems. However, the difference in *M*_o_ between single- (55 µg m^−3^ for BVOCs and 3.5 µg m^−3^ for toluene) and multi-precursor systems (14.5 µg m^−3^) complicates the comparison of measured and predicted SOA mass yields. Therefore, it is critical to assess changes in SOA mass yield as a function of *M*_o_. It is also worth noting that the same study^24^ detected non-linear effects on volatility in their simultaneous oxidation of anthropogenic and biogenic VOCs, consistent with our volatility analysis.

It is important that multi-precursor effects are studied and considered in the context of atmospherically relevant conditions. Typical oxidation conditions in ambient air resemble the MIX experiment more than the SEQ experiment because VOCs are often present as a mixture in the atmosphere. In addition, non-linear effects in the multi-precursor systems investigated in this study are likely important in environments where RO_2_· concentrations are relatively higher compared to other radicals. Nighttime is one such environment, in which concentrations of competing radicals (e.g., NO, HO_2_·, etc.) are much lower than during daytime due to the absence of photochemistry. The direct emission of NO is also minimal due to less traffic activities at night. Consequentially, RO_2_· + RO_2_· chemistry is often more pronounced at nighttime^26,59^. Particle-phase oligomerization reactions also substantially contribute to the SOA formation for nitrate radical oxidation of monoterpenes^43,50^, making the non-linear effects in multi-precursor systems potentially more relevant for nitrate radical oxidation than for photo-oxidation. Thus, if changes in SOA mass yield under simultaneous oxidation conditions are not considered, the modeled SOA mass concentration from oxidation of VOC mixtures by nitrate radicals could be inaccurate.

This work provides fundamental insights into SOA formation and properties from precursor mixtures, which is critical for understanding SOA formation in the atmosphere. While changes in α-pinene and limonene SOA mass yields during simultaneous oxidation by nitrate radicals are investigated in this study, it is possible that oxidation of different combinations of VOCs results in a similar outcome. For example, in the southeastern U.S., an enhanced isoprene SOA mass yield via nitrate radical oxidation compared with laboratory chamber experiments was observed^60^. The authors proposed that the enhancement might be attributed to a much longer lifetime of RO_2_· in ambient air than in chamber experiments, enabling oxidation intermediates to undergo isomerization and reduce volatility. As with the case of an enhancement of α-pinene SOA mass yield in our study, it is possible that cross-reactions in the gas and/or particle phase in multi-precursor systems lead to reduced volatility of isoprene oxidation products, which would result in an increase in isoprene SOA mass yield. The abundant low-volatility oxidation products from oxidation of monoterpenes in the southeastern U.S.^26,27^ may be the key ingredients for such non-linearity in isoprene SOA mass yield. It warrants further studies to investigate to what extent non-linear effects on chemical composition and volatility contribute to such increased isoprene SOA mass yield in ambient environments.

## Methods

### Chamber experiment

Laboratory chamber experiments were conducted in batch mode at the Georgia Tech Environmental Chamber facility, housing two 12 m^3^ Teflon reactors^61^. The experiments used in this study were similar to the experiments reported in Berkemeier et al.^42^ and were named APN, LIM, SEQ, and MIX, representing the type of VOC or injection order. APN and LIM represent experiments with only α-pinene or limonene as the precursor VOC, respectively. SEQ represents an experiment where α-pinene was first introduced into the chamber and oxidized, followed by the introduction and oxidation of limonene. MIX represents an experiment in which both α-pinene and limonene were oxidized simultaneously. All VOCs were oxidized by nitrate radicals.

Chamber experiments follow the procedure outlined below. Prior to chamber experiments, the chamber was flushed with zero air (Aadco, 747–14) for at least a day and a half. The chamber was conditioned to 5 °C and low humidity (RH < 5%). Ammonium sulfate seed particles were introduced into the chamber by atomizing a 0.015 M ammonium sulfate solution. This facilitated condensation of organic vapors onto existing seed particles and minimized their loss to the chamber walls. The typical seed number and volume concentrations were 26–32 × 10^3^ cm^−3^ and 20–30 × 10^9^ nm^3^ cm^−3^, respectively. Precursor VOCs were also introduced into the chamber during the same time period. A known amount of α-pinene or limonene in liquid form was transferred into a glass bulb, evaporated, and carried into the chamber by flowing zero air at 5 L min^−1^ through the bulb. Concentrations of precursor VOCs were measured by a gas chromatograph flame ionization detector using an Agilent HP-5 column (GC-FID; 7890 A, Agilent Technology Inc.). After at least three GC-FID scans showing stable VOC levels, the oxidant precursor (i.e., N_2_O_5_) was added to the chamber, marking the beginning of the experiment. The source of nitrate radicals in this study was the thermal dissociation of N_2_O_5_, which was pre-made by a reaction of NO_2_ and O_3_ in a 1.5 L flow tube^61^. The mixing ratio of NO_2_ and O_3_ was adjusted to maximize the production of N_2_O_5_ and minimize the concentration of O_3_, so that nitrate radical oxidation was the dominant VOC degradation pathway. The duration of N_2_O_5_ injection (~11 ppb min^−1^) was adjusted such that the ratio of N_2_O_5_ to initially present double bond was 4 (i.e., [N_2_O_5_]:[α-pinene] = 4:1 and [N_2_O_5_]:[limonene] = 8:1). Upon N_2_O_5_ injection, precursor VOCs quickly decayed away (no peak was present by the 1^st^ or 2^nd^ GC-FID scan after the beginning of the experiment) and NO_2_ quickly formed and stabilized. NO concentration was close to or below the detection limit (i.e., 0.4 ppb) in all the experiments. The SOA mass concentration reached the maximum typically at 120–180 min after the beginning of the experiment. For the SEQ experiment, limonene was injected into the chamber at 60 min since the start of the experiment. Immediately after the introduction of limonene, additional N_2_O_5_ was injected. Note that almost all limonene appeared to have reacted away before additional N_2_O_5_ injection due to leftover N_2_O_5_ from initial α-pinene oxidation. A summary of the experimental conditions is shown in Table 1.

### Instrumentation

A filter inlet for gases and aerosols coupled to a high-resolution time-of-flight iodide chemical ionization mass spectrometer (FIGAERO-CIMS; Aerodyne Research Inc.) was deployed to measure chemical composition of oxidized organic species in both gases and particles in an hourly basis^54,56,62^. Thermal desorption profiles measured by FIGAERO-CIMS (i.e., signal vs. temperature) also allowed for inferring the volatility of SOA. The operational details of the instrument were the same as reported in a previous study^46^. Briefly, particles were collected on a Teflon filter (Zefluor, 2 µm pore size) at 1.67 L min^−1^ for 30 min via a dedicated quarter-inch stainless steel tubing and were subsequently desorbed with heated dry N_2_ to be detected by iodide-adduct CIMS. The desorbing N_2_ temperature was ramped from 25 °C to 175 °C during first 15 min, kept at 190 °C for 10 min, and cooled for 5 min before the next particle collection period resumed. Raw data were averaged to 10-s intervals and then analyzed using Tofware 2.5.11 based on Igor Pro 6.37. Reported signals (count s^−1^) were normalized to 10^6^ of reagent ions (i.e., I^−^ + H_2_O·I^−^). Compounds detected as iodide adducts were considered. The desorption cycle immediately before the start of reaction served as the baseline (background signal). All the data presented in this work have been corrected for background. A FIGAERO-CIMS mass spectrum refers to the average of two thermal desorption cycles, closest to the time when the maximum SOA mass concentration was observed.

A high-resolution time-of-flight aerosol mass spectrometer (AMS; Aerodyne Research Inc.) measured the chemical composition of bulk non-refractory submicron aerosol^63,64^. Owing to flash vaporization of aerosol and hard ionization (i.e., electron impact ionization), ions were often detected as fragments of compounds. It was, however, still able to provide a unique fingerprint of SOA from each system. AMS sampled the chamber air from a dedicated quarter-inch stainless steel tubing at a flow rate of 0.08 L min^−1^. Raw data were saved every minute. SQUIRREL v1.57I and PIKA v1.16I based on Igor Pro 6.37 were used to analyze AMS data.

A scanning mobility particle sizer (SMPS; TSI Inc.), composed of a differential mobility analyzer 3080 (TSI Inc.) and a condensation particle counter 3075 (TSI Inc.), measured aerosol size distributions and volume concentrations. SMPS sampled the chamber air from a dedicated quarter-inch stainless steel tubing at a flow rate of 0.3 L min^−1^. The sheath flow rate was set at 2 L min^−1^. The scanning particle size range was from 17.5 to 982.2 nm. Each size distribution measurement cycle lasted 6 min. A cavity attenuated phase shift monitor (CAPS; Aerodyne Research Inc.)^65^, a NO_x_ monitor (Thermo 42C), and a UV absorption O_3_ analyzer (Teledyne T400) measured NO_2_, NO, and O_3_, respectively.

### Calculation of α-pinene and limonene SOA mass yields in the SEQ and MIX experiments

Concentrations of reacted precursor VOCs (ΔVOC) were measured by GC-FID. SMPS volume concentrations were corrected for particle wall loss (PWL) using size-dependent, first-order PWL coefficients, determined by a separate PWL experiment^61^. SOA mass concentrations were calculated by PWL-corrected SMPS volume concentrations multiplied by SOA density (1.46 and 1.64 µg m^−3^ for α-pinene and limonene SOA, respectively)^44,53^. The uncertainty of SMPS volume concentrations was 5%, and the uncertainty of the SOA density was 2% based on the repeated measurement of ammonium nitrate seed density during routine AMS ionization efficiency calibrations. The uncertainties of SOA mass concentrations and mass yields were calculated based on the standard error propagation method.

Determining the SOA mass yield of an individual VOC in a multi-precursor system is not trivial. For the SEQ experiment, α-pinene was first oxidized by nitrate radicals without the presence of limonene. Thus, α-pinene SOA mass yield in the SEQ experiment could be treated as a single-precursor system, as with the APN experiment. However, limonene SOA mass yield in the SEQ experiment and both α-pinene and limonene SOA mass yield in the MIX experiments cannot be treated as a single-precursor system.

As discussed in the results section in the main text, AMS mass spectra observed in the SEQ and MIX experiments could be well predicted as linear combinations of the mass spectra in the APN and LIM experiments. In order to apportion the fractional contribution of α-pinene and limonene oxidation products to the total SOA mass concentration, we used multiple linear regression analysis on 30-min averaged AMS mass spectra during the peak SOA mass period in the APN, LIM, SEQ, and MIX experiments. In the SEQ experiments, α-pinene and limonene oxidation products contributed to the total SOA mass concentration by 47–48% and 52–53%, respectively. These mass fractions corresponded to 30.1–30.4 µg m^−3^ and 32.3–34.8 µg m^−3^ for Δ*M*_o,α-pinene_ and Δ*M*_o,limonene_ in the SEQ experiments, respectively (Supplementary Table 1). On the other hand, in the MIX experiments, α-pinene and limonene oxidation products contributed to the SOA mass concentration by 56–57% and 43–44%, respectively. These mass fractions corresponded to 42.9–46.0 µg m^−3^ and 32.8–35.8 µg m^−3^ for Δ*M*_o,α-pinene_ and Δ*M*_o,limonene_ in the MIX experiments, respectively (Supplementary Table 1).

The validity of the apportionment method based on the difference in AMS mass spectra was evaluated using the SEQ experiment. In the SEQ experiment, there was a step-wise increase in the SOA mass concentration upon oxidation of each VOC. The first increase corresponded to the α-pinene SOA formation. The second increase represented limonene oxidation products, in addition to further condensation of α-pinene oxidation products depending on whether α-pinene and limonene SOA are mixed or not. For example, for the SEQ-1 experiment, upon oxidation of limonene in the presence of α-pinene oxidation products, SOA mass concentration increased by 36.7 µg m^−3^. Assuming that α-pinene and limonene oxidation products formed an ideal solution, this increase in SOA mass concentration was attributed not only to limonene SOA formation but also to the further condensation of already existing gaseous α-pinene oxidation products due to increase in *M*_o_^8^. The further condensation of gaseous α-pinene oxidation products could be quantified if α-pinene SOA mass yield was known at the *M*_o_ of interest. The use of α-pinene SOA mass yield curve enabled us to estimate that 5.2 µg m^−3^ out of the observed 36.7 µg m^−3^ was attributed to the further condensation of gaseous α-pinene oxidation products. Thus, Δ*M*_o,α-pinene_ and Δ*M*_o,limonene_ could be calculated as follows:

1 $$\varDelta {M}_{{{{{{\rm{o}}}}}},{{{{{\rm{\alpha }}}}}}-{{{{{\rm{pinene}}}}}}}=25.7({{{{{\rm{i}}}}}}{{{{{\rm{.e}}}}}}.,\, \varDelta {M}_{{{{{{\rm{o}}}}}},{{{{{\rm{observed}}}}}}}\,{{{{{\rm{in}}}}}}\,{{{{{\rm{first}}}}}}\,{{{{{\rm{increase}}}}}})+5.2=30.9\,\mu {{{{{\rm{g}}}}}}\,{{{{{\rm{m}}}}}}^{-3}$$

2 $$\varDelta {M}_{{{{{{\rm{o}}}}}},{{{{{\rm{limonene}}}}}}}=36.7({{{{{\rm{i}}}}}}{{{{{\rm{.e}}}}}}.,\, \Delta {M}_{{{{{{\rm{o}}}}}},{{{{{\rm{observed}}}}}}}\,{{{{{\rm{in}}}}}}\,{{{{{\rm{second}}}}}}\,{{{{{\rm{increase}}}}}})-5.2=31.5\,\mu {{{{{\rm{g}}}}}}\,{{{{{\rm{m}}}}}}^{-3}$$

In contrast, under the assumption of phase separation, α-pinene and limonene SOA were considered not to interact with each other and, thus, *M*_o_ was no longer the total but the individual SOA mass concentration. Also, the increase of SOA mass concentration upon limonene oxidation solely came from the condensation of limonene oxidation products. Thus, Δ*M*_o,α-pinene_ and Δ*M*_o,limonene_ are the same as the first and second increases of the SOA mass concentration, respectively. This increases the contribution of limonene oxidation products and decreased that of α-pinene oxidation products to the total SOA mass concentration, compared to the ideal mixing scenario.

The Δ*M*_o,α-pinene_ and Δ*M*_o,limonene_ values obtained based on AMS mass spectra analysis were comparable to those derived above using the step-wise SOA mass concentration increase with either assumption of ideal mixing or phase separation (Supplementary Table 4). This result supported the validity of the SOA mass apportionment approach based on the difference in AMS mass spectra.

### Calculation of volatility distribution of oxidation products in the APN and LIM experiments

FIGAERO-CIMS employed thermal desorption technique to measure particle-phase species. The temperature at which a compound showed the maximum signal (*T*_max_) was shown to correlate with its saturation mass concentration (C^*^)^55,56,66^. Using compounds with known saturation mass concentrations, volatility calibration was performed. The calibrants used in this study included malonic acid (C_3_H_3_O_4_), succinic acid (C_4_H_6_O_4_), meso-erythritol (C_4_H_10_O_4_), xylitol (C_5_H_12_O_5_), levoglucosan (C_6_H_10_O_5_), suberic acid (C_8_H_14_O_4_), mannitol (C_6_H_14_O_6_), azelaic acid (C_9_H_16_O_4_), sebacic acid (C_10_H_18_O_4_), trimesic acid (C_9_H_6_O_6_), tridecanoic acid (C_13_H_26_O_2_), cyclohexane-1,3,5-tricarboxylic acid (C_e_H_12_O_6_), dodecanedioic acid (C_12_H_22_O_4_), dipentaerythritol (C_10_H_22_O_7_), palmitic acid (C_16_H_32_O_2_), stearic acid (C_18_H_36_O_2_), and benenic acid (C_22_H_44_O_2_). The stock solutions at ~0.1 g mL^−1^ in HPLC-grade acetone were prepared and were diluted immediately before conducting calibration experiments such that the working solution contained the mixture of organics at 0.002 and 0.01 g mL^−1^. Using a 10 µL gas-tight syringe (Hamilton), 1–5 µL of the working solution was deposited on a pre-conditioned Teflon filter (Zefluor) that resulted in 2–50 ng for each compound on the filter. Calibration used the same temperature ramping (15 min) setting as the chamber experiments. Average *T*_max_ observed for the calibration compounds spanned from 34 to 155 °C, which corresponded to log_10_(C^*^ [µg m^−3^]) of 2 to −8 (Supplementary Fig. 6).

In order to derive the volatility distribution for particle-phase compounds from FIGAERO-CIMS thermogram, information regarding the peak shape was also required. We followed the approach outlined in Stark et al.^55^. Basis function for a representative peak shape was composed of a Gaussian function and a Lorentzian function that was used to simulate the right-hand side tailing. The inclusion of 25% uncertainty in each coefficient of the peak shape function could cover the majority of the variation in the peak shape from different compounds (Supplementary Fig. 7).

Measured sum thermograms in the APN-2 and LIM-2 experiments were fitted using the constrained peak shape using the non-linear least square method. Each coefficient of peak shape function was allowed to vary by up to 25%, to be consistent with the variation observed in the calibrants. Often the bulk SOA thermogram could not be well-fitted with a single peak. Thus, the additional peak was allowed to be added one by one in an iterative manner until the improvement in the fractional residual was less 10%. The thermogram in the APN experiment required two peaks, whereas that in the LIM experiment needed five peaks (Supplementary Fig. 8). *T*_max_ of each fitted peak corresponded to C^*^ by the aforementioned calibration curve. Area under the curve of each peak was related to the fractional contribution from each peak to total signal and thus particle-phase volatility distribution. Conversion of particle-phase volatility distribution into both-phase (gas- and particle-phases) volatility distribution required the following equation:

3 $${{{{\mathrm{F}}}}}{{{{{\mathrm{C}}}}}}_{{{{{\mbox{g}}}}}+{{{{\mathrm{p}}}}}, \, i}=\frac{{{{{\mathrm{F}}}}}{{{{{\mathrm{C}}}}}}_{{{{{\mathrm{p}}}}}, \, i}}{{F}_{{{{{\mathrm{p}}}}},\, i}}=\frac{{{{{\mathrm{F}}}}}{{{{{\mathrm{C}}}}}}_{{{{{\mathrm{p}}}}}, \, i}}{\left(\frac{1}{1+\frac{{{C}^{*}}_{i}}{{M}_{{{{{\mathrm{O}}}}}}}}\right)}$$

where FC_g+p,*i*_ and FC_p,*i*_ were fractional contributions of peak *i* to total signal in both phases and particle phase, *F*_p,*i*_ was particle fraction of peak *i*, C^*^_i_ was the saturation mass concentration of peak *i*. Once the fractional contributions of peak *i* to total signal in both phases were found, the yield data points from the APN-2 and LIM-2 experiments were used as constraints to convert the relative contributions to particle yields in a unit of µg m^−3^.

### Multiple linear regression analysis for comparison between single- and multi-precursor systems

In order to evaluate whether FIGAERO-CIMS and AMS mass spectra and FIGAERO-CIMS thermal desorption profiles of the SEQ and MIX experiments could be successfully predicted as linear combinations of those derived from the APN and LIM experiments, we performed multiple linear regression analysis using the following equation.

4 $$\left(\begin{array}{c}{S}_{{{{\mathrm{SEQ}}}} \ {\mathrm {or}} \ {\mathrm {MIX}}, 1}\\ \vdots \\ {S}_{{{{\mathrm{SEQ}}}} \ {\mathrm {or}} \ {\mathrm {MIX}}, i}\\ \vdots \\ {S}_{{{{\mathrm{SEQ}}}} \ {\mathrm {or}} \ {\mathrm {MIX}}, n}\end{array}\right)=\left(\begin{array}{cc}{S}_{{{{{\mathrm{APN}}}}}, 1} & {S}_{{{{{\mathrm{LIM}}}}}, 1}\\ \vdots & \vdots \\ {S}_{{{{{\mathrm{APN}}}}}, i} & {S}_{{{{{\mathrm{LIM}}}}}, i}\\ \vdots & \vdots \\ {S}_{{{{{\mathrm{APN}}}}},n} & {S}_{{{{{\mathrm{LIM}}}}},n}\end{array}\right)\left(\begin{array}{c}{x}_{{{{{\mathrm{APN}}}}}}\\ {x}_{{{{{\mathrm{LIM}}}}}}\end{array}\right)+\left(\begin{array}{c}{r}_{1}\\ \vdots \\ {r}_{i}\\ \vdots \\ {r}_{n}\end{array}\right)$$

*S*_SEQ or MIX,__*i*_ represented a signal at nominal *m/z i* or at desorption temperature *i* from either SEQ or MIX experiment, *S*_APN,*i*_ and *S*_LIM,*i*_ indicated a signal at nominal *m/z i* or at desorption temperature *i* from the APN and LIM experiments, respectively, *x*_APN_ and *x*_LIM_ were the regression coefficients obtained via multiple linear regression analysis and were indicative of the fraction of the APN and LIM experiments used for prediction, and *r*_*i*_ was a residual at a nominal *m/z i* or at desorption temperature *i*. The obtained coefficients were then used to predict signals in the SEQ and MIX experiments at nominal *m/z i* or at desorption temperature *i* and were compared with measured signals.

In preparing for multiple linear regression analysis, FIGAERO-CIMS and AMS mass spectra obtained in the APN, LIM, SEQ, and MIX experiments were normalized to the sum of 1. To facilitate an easier comparison, exact *m/z* of species were converted to nominal *m/z*. The temperature ramping profiles in all the experiments were close enough that it was reasonable to compare the signals measured at the same time period of cycle. Omitting the first data point due to signal fluctuation induced by the actuator position change of the FIGAERO-CIMS, a total of 89 data points during the FIGAERO-CIMS temperature ramping period were used. Averages of two thermal desorption profiles closest to the time when the maximum SOA mass concentration reached were used. Thermal desorption profiles were then normalized to the sum of 1, as with FIGAERO-CIMS and AMS mass spectra of SOA.

## Supplementary information

Supplementary Information

## Data Availability

The chamber experiment data used in this study are available in the Index of Chamber Atmospheric Research in the United States (ICARUS) database (https://icarus.ucdavis.edu/experimentset/256).

## Code availability

We used commercial software (Tofware 2.5.11, SQUIRREL v1.57I, and PIKA v1.16I) for data analysis. Other codes used in this study are available upon request from the corresponding author.
