# Supplementary material for: Non-linear effects of secondary organic aerosol formation and properties in multi-precursor systems
Source: Nat Commun. 2022 Dec 22;13:7883. doi: 10.1038/s41467-022-35546-1 (PMC9780343; doi:10.1038/s41467-022-35546-1)
Supplement: Supplementary file 1 — Supplementary Information [file 41467_2022_35546_MOESM1_ESM.pdf]

Supplementary Information for

Non-linear effects of secondary organic aerosol formation and properties in multi-precursor systems

Masayuki Takeuchi,[1] *Thomas Berkemeier,*[2]† *Gamze Eris,*[2] *Nga Lee Ng*[1,2,3]*

[1]School of Civil and Environmental Engineering, Georgia Institute of Technology, Atlanta, GA, 30332, USA.

[2]School of Chemical and Biomolecular Engineering, Georgia Institute of Technology, Atlanta, GA, 30332, USA

[3]School of Earth and Atmospheric Sciences, Georgia Institute of Technology, Atlanta, GA, 30332, USA.

†Now at: Multiphase Chemistry Department, Max Planck Institute for Chemistry, Mainz, Germany

*Corresponding author: Nga Lee Ng (ng@chbe.gatech.edu)

Total Pages: 17
Number of Notes: 1
Number of Figures: 9
Number of Tables: 4

Supplementary Note 1: Assignment of chemical formulae beyond the *m/z* of the largest mass calibrant

An accurate *m/z* calibration is essential to correctly assign molecular formulae to peaks observed in a mass spectrum. During the experiments presented in this study, there was a lack of reliable *m/z* calibrants beyond *m/z* 381 ($I_2{\cdot}I^-$), though high intensity ions were detected up to *m/z* ~900 (Fig. 2). Since a substantial error in *m/z* calibration at such high *m/z* was expected, we performed a follow-up experiment using perfluoropentanoic and perfluoroheptanoic acids (Sigma-Aldrich) to evaluate the magnitude of *m/z* calibration errors at high *m/z*. Chemical formulae of potential ions to be detected in CIMS using these chemicals were known[1]. We identified seven ions to compare the measured *m/z* with their exact *m/z*. Perfluoropentanoic acid (C_4F_9COOH) produced three prominent ions: $(C_4F_9COOH)C_4F_9COO^-$ at *m/z* 527, $(C_4F_9COOH)_2{\cdot}I^-$ at *m/z* 655, and $(C_4F_9COOH)_2C_4F_9COO^-$ at *m/z* 791. On the other hand, perfluoroheptanoic acid ($C_6F_{13}COOH$) was detected as $(C_6F_{13}COOH){\cdot}I^-$ at *m/z* 491, $(C_6F_{13}COOH)C_6F_{13}COO^-$ at *m/z* 727, $(C_6F_{13}COOH)_2{\cdot}I^-$ at *m/z* 855, and $(C_6F_{13}COOH)_2C_6F_{13}COO^-$ at *m/z* 1091. To evaluate the magnitude of *m/z* calibration errors, we used the same set of mass calibrants used in the chamber experiments (i.e., I^-, $H_2O{\cdot}I^-$, $CHOOH{\cdot}I^-$, $HNO_3{\cdot}I^-$, $I{\cdot}I^-$, $I_2{\cdot}I^-$) and compared a peak *m/z* vs. exact *m/z* of the expected ions. Errors were small up to *m/z* 500 but began to greatly deviate beyond (Supplementary Fig. 9). Given the expected magnitude of *m/z* calibration errors and knowledge of volatile organic compound (VOC) degradation chemistry, it was possible to narrow down the chemical formulae of detected ions at high *m/z*.

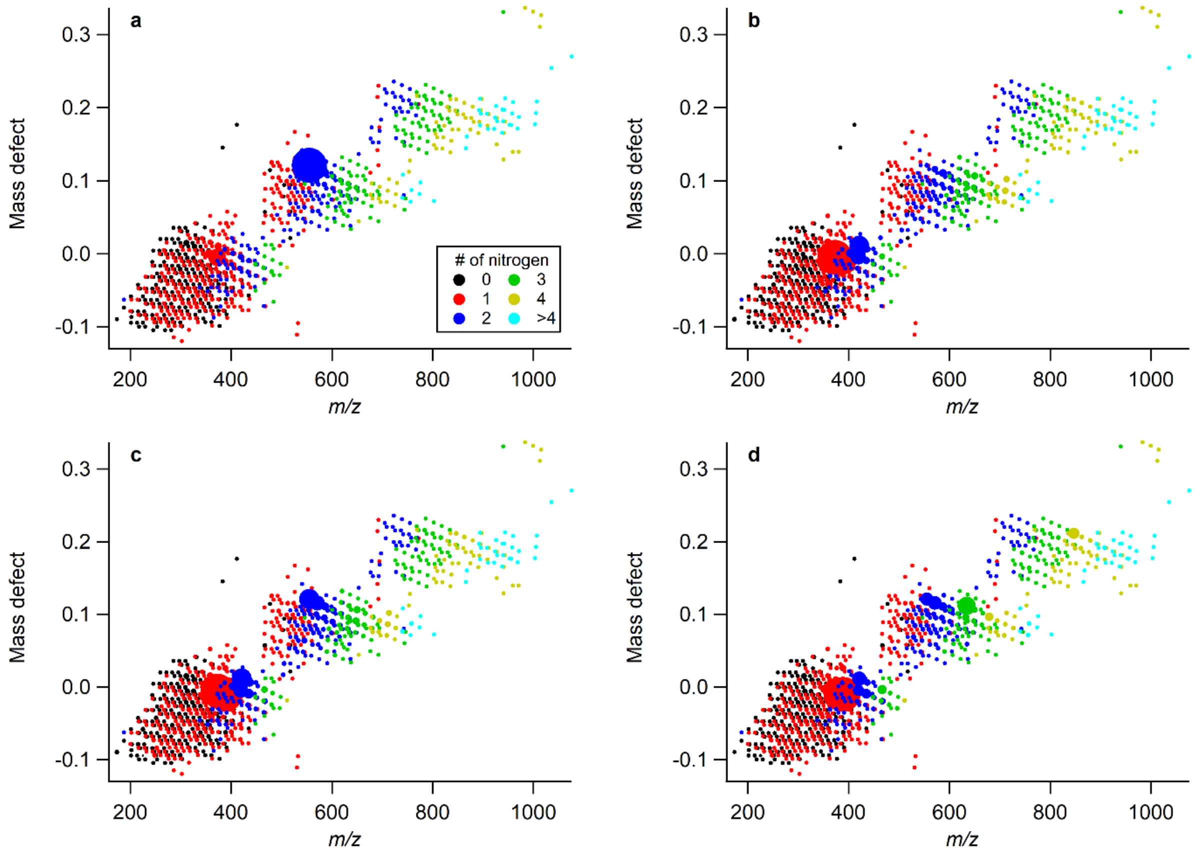

Supplementary Fig. 1. Mass defect plots. Mass defect plots for a filter inlet for gases and aerosols coupled to a chemical ionization mass spectrometer (FIGAERO-CIMS) data in **a** pure α-pinene (APN), **b** pure limonene (LIM), **c** sequential oxidation (SEQ), and **d** simultaneous oxidation (MIX) experiments. Markers are colored by the number of nitrogen and the size of markers is proportional to signal intensity.

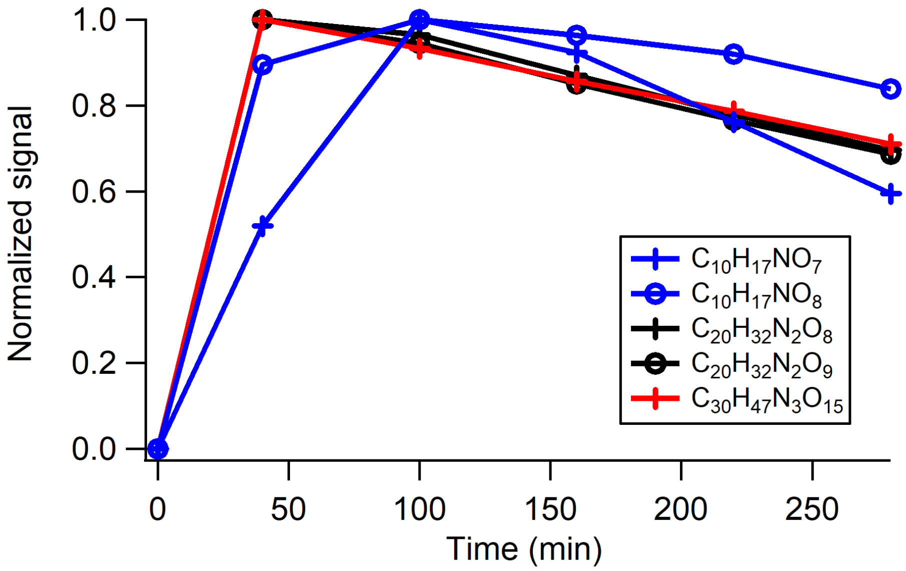

Supplementary Fig. 2. A filter inlet for gases and aerosols coupled to a chemical ionization mass spectrometer (FIGAERO-CIMS) time series data of selected particle-phase species in the pure α-pinene (APN) experiment.

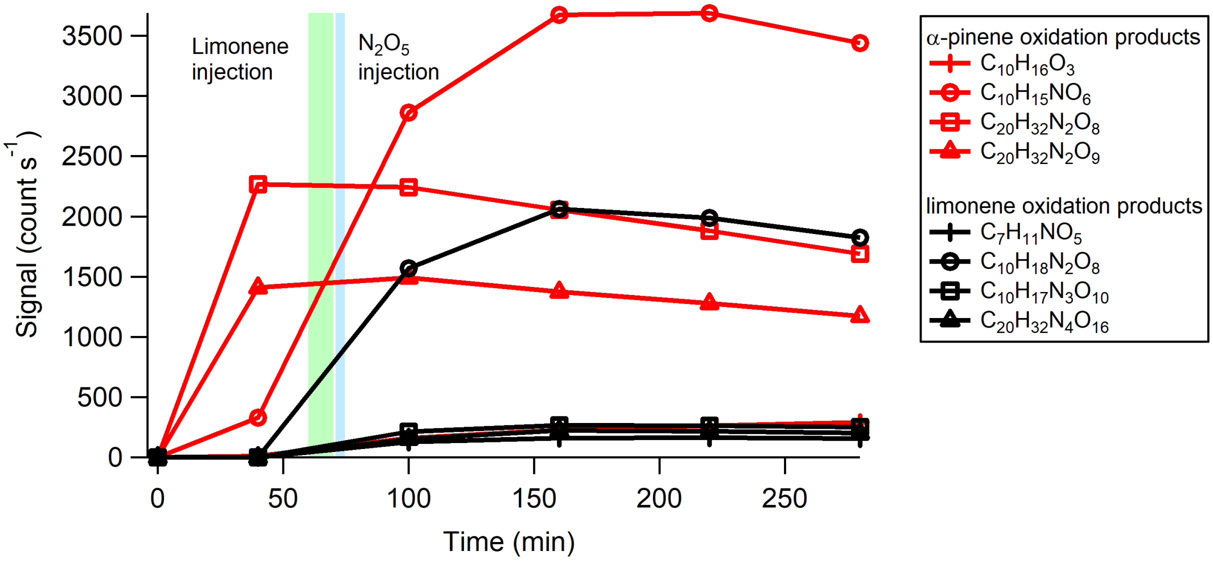

Supplementary Fig. 3. A filter inlet for gases and aerosols coupled to a chemical ionization mass spectrometer (FIGAERO-CIMS) time series data of particle-phase species in the sequential oxidation (SEQ) experiment. Compounds are chosen based on the major oxidation products observed in the pure α-pinene (APN) and pure limonene (LIM) experiments. The period of limonene injection is shaded in green, whereas the N_2O_5 injection period is shown in blue.

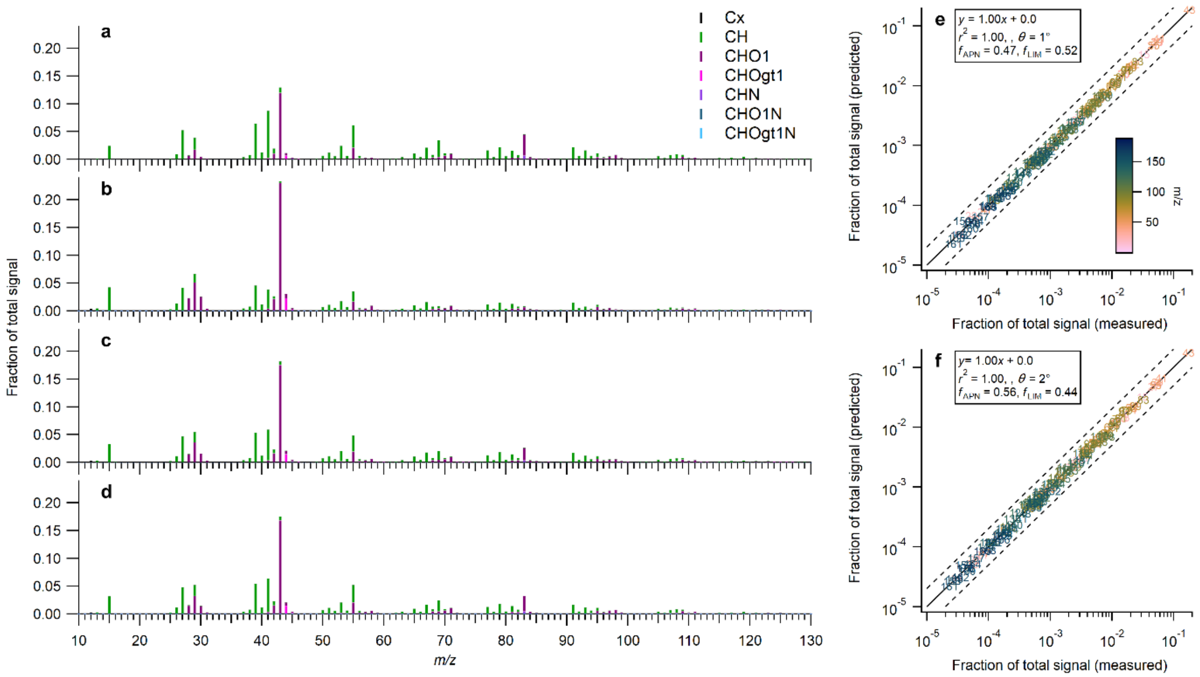

Supplementary Fig. 4. Mass spectra of an aerosol mass spectrometer (AMS) and their comparison between single- and multi-precursor systems. AMS mass spectra in **a** pure α-pinene (APN), **b** pure limonene (LIM), **c** sequential oxidation (SEQ), and **d** simultaneous oxidation (MIX) experiments. Colors indicate the type of family: carbon (Cx), hydrocarbon (CH), carbonaceous with one oxygen (CHO1), carbonaceous with greater than one oxygen (CHOgt1), carbonaceous with one nitrogen (CHN), carbonaceous with one nitrogen and one oxygen (CHO1N), and carbonaceous with one nitrogen and greater than one oxygen (CHOgt1N). Comparison of AMS mass spectrum obtained in **e** SEQ and **f** MIX experiments with those predicted based on linear combinations of APN and LIM experiments. Solid line indicates 1:1, while dashed lines represent a deviation of a factor of 2. Data points are numbered and colored by *m/z*. θ is a spectral contrast angle, and f_{APN} and f_{LIM} indicate the signal fractions of APN and LIM experiments contributing to secondary organic aerosol estimated via multiple linear regression analysis, respectively.

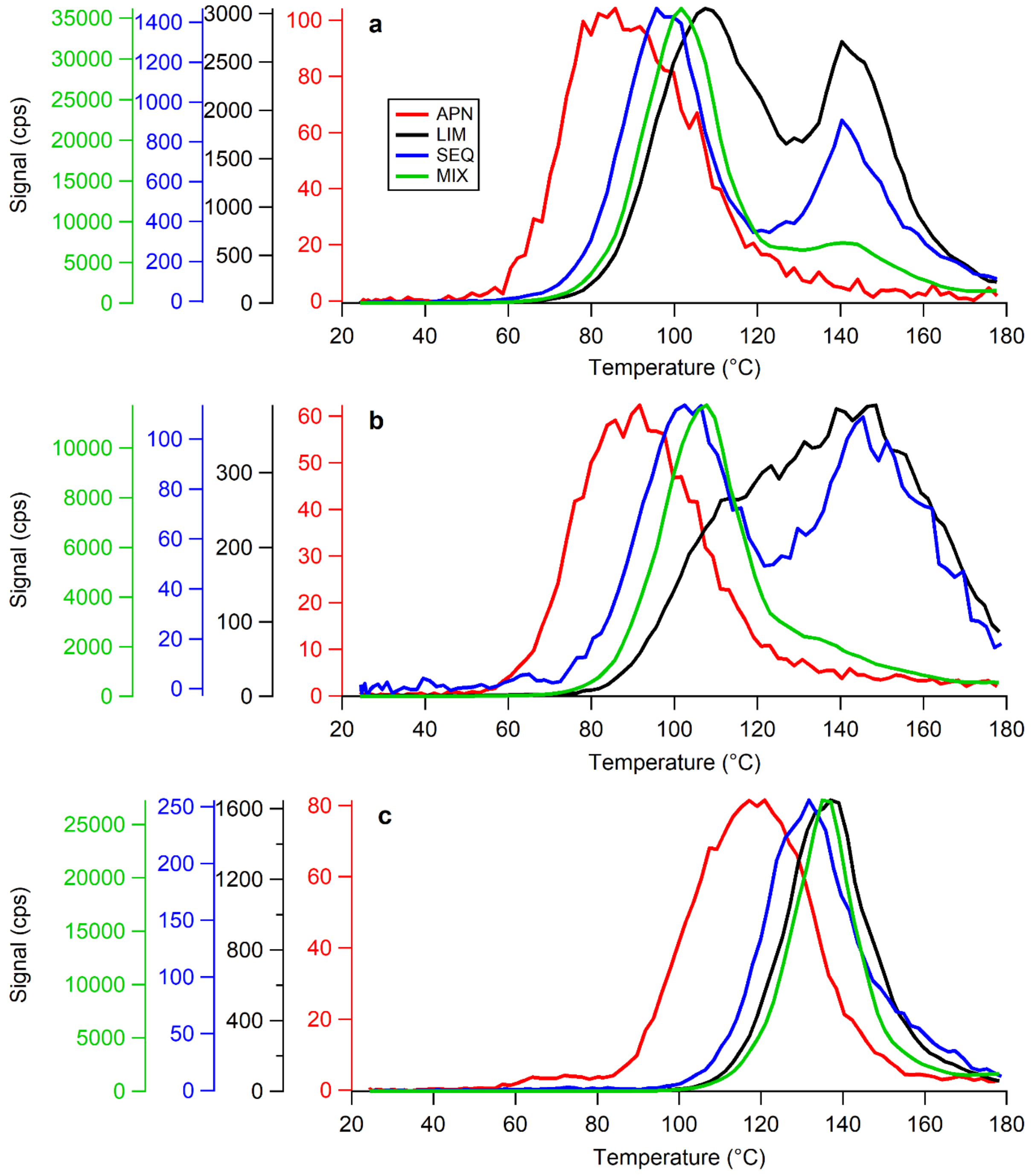

Supplementary Fig. 5. Thermogram of characteristic ions. A filter inlet for gases and aerosols coupled to a chemical ionization mass spectrometer (FIGAERO-CIMS) thermogram of **a** $C_{20}H_{33}N_3O_{12}\cdot I^-$, **b** $C_{20}H_{32}N_4O_{14}\cdot I^-$, and **c** $C_{30}H_{48}N_4O_{16}\cdot I^-$ observed in pure α-pinene (APN), pure limonene (LIM), sequential oxidation (SEQ), and simultaneous oxidation (MIX) experiments.

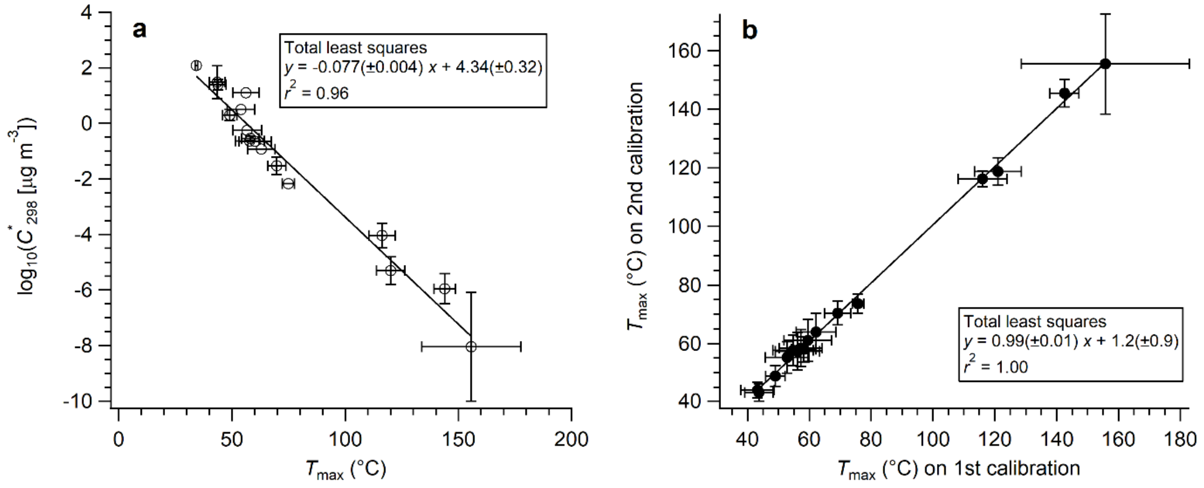

Supplementary Fig. 6. Volatility calibration curve of a filter inlet for gases and aerosols coupled to a chemical ionization mass spectrometer (FIGAERO-CIMS). a Volatility calibration curve of FIGAERO-CIMS. Open circles and error bars are mean values and standard deviation of two calibrations, conducted at the beginning and end of the entire study period, respectively. The line represents a total least-squares fit. **b** Comparison of two calibrations showing consistent T_{max} between the two calibrations. Error bars represent standard deviations of four or five scans in each calibration.

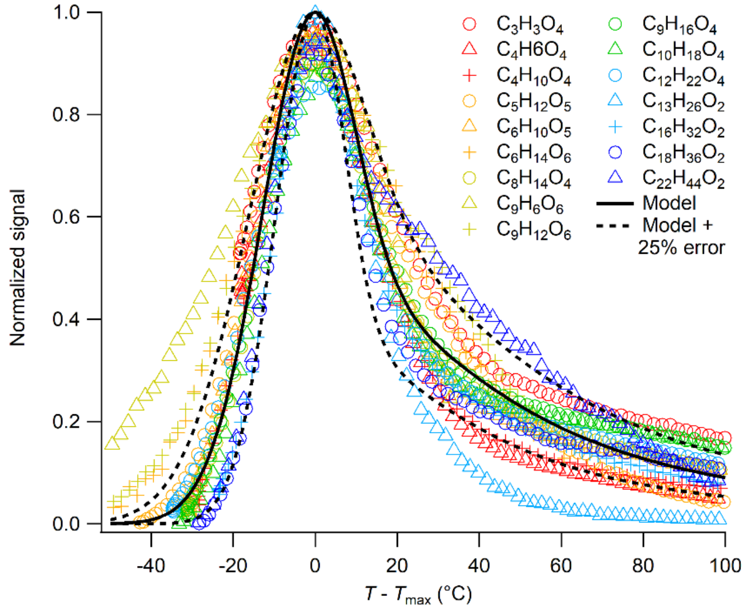

Supplementary Fig. 7. Basis function for a representative peak shape used in thermogram fitting.

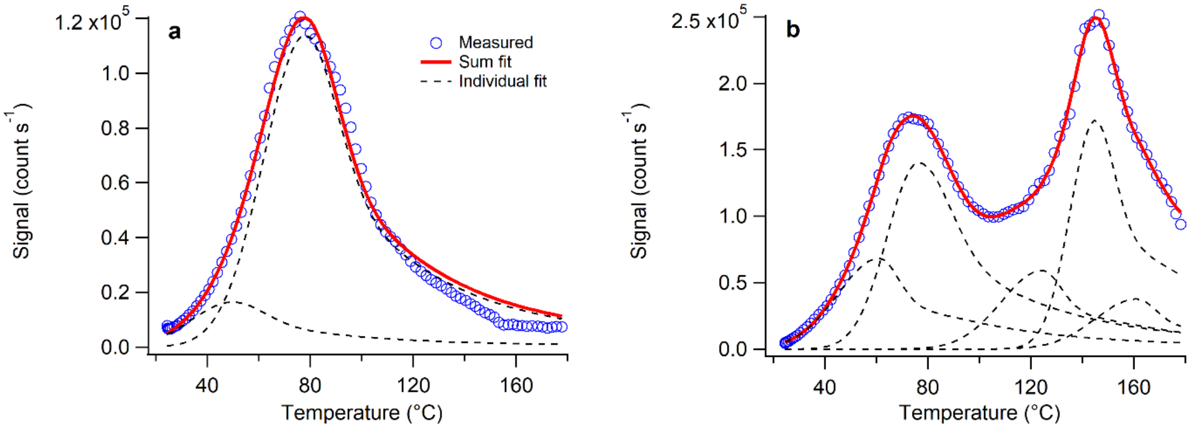

Supplementary Fig. 8. Sum thermograms. Sum thermograms and peak fits in **a** pure α-pinene (APN) and **b** pure limonene (LIM) experiments.

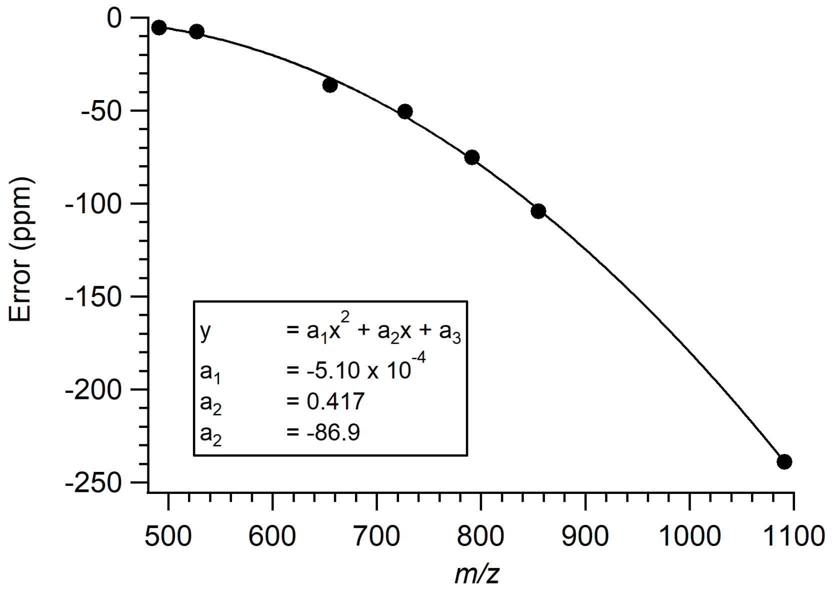

Supplementary Fig. 9. Error in a filter inlet for gases and aerosols coupled to a chemical ionization mass spectrometer (FIGAERO-CIMS) *m/z* calibration as a function of *m/z*. Error is calculated using perfluoropentanoic and perfluoroheptanoic acids as the ratio of difference between measured and expected *m/z* to expected *m/z*.

Supplementary Table 1. List of secondary organic aerosol (SOA) mass yields. Experiment names are based on the type of volatile organic compound (VOC) or order of injection. ΔVOC represents the amount of precursor VOC(s) oxidized during experiments and the associated errors are based on the calibration of a gas chromatograph-flame ionization detector (GC-FID). ΔM_o represents the mass concentration of organics formed from either α-pinene or limonene and the associated errors are based on the uncertainties of a scanning mobility particle sizer (SMPS) and SOA density. Y_{SOA} represents SOA mass yield, and M_o indicates the organic mass concentration present in the system, corresponding to Y_{SOA}. Errors for Y_{SOA} are calculated by the standard error propagation. Errors for M_o are calculated in the same manner as ΔM_o.

Exp.	Experiment variant		α-pinene				limonene			
			ΔVOC (μg m^{-3})	ΔM_o (μg m^{-3})	Y_{SOA}	M_o (μg m^{-3})	ΔVOC (μg m^{-3})	ΔM_o (μg m^{-3})	Y_{SOA}	M_o (μg m^{-3})
APN-1	Pure α-pinene, low conc.		135.1 ± 9.5	26.6 ± 1.4	0.20 ± 0.02	26.6 ± 1.4	-	-	-	-
APN-2	Pure α-pinene, med conc.		291.4 ± 11.1	61.8 ± 3.3	0.21 ± 0.01	61.8 ± 3.3	-	-	-	-
APN-3	Pure α-pinene, high conc.		449.4 ± 13.5	105.2 ± 5.7	0.23 ± 0.01	105.2 ± 5.7	-	-	-	-
LIM-1	Pure limonene, low conc.		-	-	-	-	24.3 ± 1.9	37.3 ± 2.0	1.53 ± 0.15	37.3 ± 2.0
LIM-2	Pure limonene, med conc.		-	-	-	-	62.0 ± 3.0	89.1 ± 4.8	1.44 ± 0.10	89.1 ± 4.8
LIM-3	Pure limonene, high conc.		-	-	-	-	89.1 ± 3.6	136.6 ± 7.4	1.53 ± 0.10	136.6 ± 7.4
LIM-4	Pure limonene, low conc., oxidant-first		-	-	-	-	30.1 ± 2.5	37.2 ± 2.0	1.23 ± 0.12	37.2 ± 2.0
LIM-5	Pure limonene, low conc., oxidant-first		-	-	-	-	30.1 ± 2.5	36.6 ± 2.0	1.22 ± 0.12	36.6 ± 2.0
SEQ-1	Sequential oxidation	(Ideal mixing)	142.6 ± 6.1	30.1 ± 1.6	0.21 ± 0.01	62.4 ± 2.4	28.4 ± 2.8	32.3 ± 1.7	1.14 ± 0.13	62.4 ± 2.4
SEQ-2	Sequential oxidation		149.4 ± 6.1	30.4 ± 1.6	0.20 ± 0.01	65.2 ± 3.5	32.6 ± 3.3	34.8 ± 1.9	1.07 ± 0.12	65.2 ± 3.5
MIX-1	Simultaneous oxidation		141.1 ± 9.5	46.0 ± 2.5	0.33 ± 0.03	81.7 ± 4.4	29.2 ± 2.5	35.8 ± 1.9	1.23 ± 0.12	81.7 ± 4.4
MIX-2	Simultaneous oxidation		138.6 ± 9.5	42.9 ± 2.3	0.31 ± 0.03	75.7 ± 4.1	27.9 ± 1.5	32.8 ± 1.8	1.18 ± 0.09	75.7 ± 4.1
SEQ-1	Sequential oxidation	(Phase separation)	142.6 ± 6.1	30.1 ± 1.6	0.21 ± 0.01	30.1 ± 1.6	28.4 ± 2.8	32.3 ± 1.7	1.14 ± 0.13	32.3 ± 1.7
SEQ-2	Sequential oxidation		149.4 ± 6.1	30.4 ± 1.6	0.20 ± 0.01	30.4 ± 1.6	32.6 ± 3.3	34.8 ± 1.9	1.07 ± 0.12	34.8 ± 1.9
MIX-1	Simultaneous oxidation		141.1 ± 9.5	46.0 ± 2.5	0.33 ± 0.03	46.0 ± 2.5	29.2 ± 2.5	35.8 ± 1.9	1.23 ± 0.12	35.8 ± 1.9
MIX-2	Simultaneous oxidation		138.6 ± 9.5	42.9 ± 2.3	0.31 ± 0.03	42.9 ± 2.3	27.9 ± 1.5	32.8 ± 1.8	1.18 ± 0.09	32.8 ± 1.8

Supplementary Table 2. Coefficients for the volatility basis set (VBS[2]).

	C^* (μg m^{-3})		
	1	10	100
α-pinene+NO_3·	0.18	0.00	0.11
limonene+NO_3·	1.00	0.59	0.00

Supplementary Table 3. Overall fates of peroxy radical ($RO_2^{\cdot}$) in four variants of chamber experiments. The relative contribution of $RO_2^{\cdot}$ loss pathways is calculated using kinetic flux analysis in a box model with gas-phase chemistry based on the master chemical mechanism (MCM).[3] $RO_2^{\cdot}$ are not allowed to partition into the particle phase in this model.

Expt.	$RO_2^{\cdot}$ fate			
	+ $RO_2^{\cdot}$	+ $NO_3^{\cdot}$	+ $HO_2^{\cdot}$	+ NO
APN-2	0.45	0.55	0.00	0.00
LIM-2	0.38	0.54	0.07	0.00
SEQ	0.49	0.51	0.01	0.00
MIX	0.46	0.53	0.00	0.00

Supplementary Table 4. Comparison of calculated mass concentration of organics (ΔM_o) in the sequential oxidation (SEQ) experiments. AMS MS difference represents the approach in which the mass fractional contributions of α-pinene and limonene oxidation products to the total secondary organic aerosol (SOA) mass concentration are calculated based on the difference in their aerosol mass spectrometer (AMS) mass spectra. Well-mixed refers to the method in which ΔM_o are calculated based on the step-wise increase in the SOA mass concentration upon oxidation of each precursor volatile organic compound (VOC). α-pinene and limonene SOA are assumed to mix ideally. Phase-separated is the same approach as well-mixed, except that α-pinene and limonene SOA are assumed to be phase-separated and thus do not interact with each other. Errors for ΔM_o are based on the uncertainties of a scanning mobility particle sizer (SMPS) and SOA density.

Approach	SEQ-1		SEQ-2	
	$\Delta M_{o,\alpha\text{-pinene}}$ (μg m^{-3})	$\Delta M_{o,\text{limonene}}$ (μg m^{-3})	$\Delta M_{o,\alpha\text{-pinene}}$ (μg m^{-3})	$\Delta M_{o,\text{limonene}}$ (μg m^{-3})
AMS MS difference	30.0 ± 1.6	32.3 ± 1.7	30.4 ± 1.6	34.8 ± 1.9
Well-mixed	30.9 ± 1.7	31.5 ± 2.9	32.6 ± 1.8	38.6 ± 2.1
Phase-separated	25.7 ± 1.4	36.7 ± 2.0	26.6 ± 1.4	32.6 ± 3.9

References

1 Heinritzi, M. *et al.* Characterization of the mass-dependent transmission efficiency of a CIMS. *Atmos Meas Tech* **9**, 1449-1460, doi:10.5194/amt-9-1449-2016 (2016).

2 Donahue, N. M., Robinson, A. L., Stanier, C. O. & Pandis, S. N. Coupled partitioning, dilution, and chemical aging of semivolatile organics. *Environ Sci Technol* **40**, 2635-2643, doi:10.1021/es052297c (2006).

3 Berkemeier, T., Takeuchi, M., Eris, G. & Ng, N. L. Kinetic modeling of formation and evaporation of secondary organic aerosol from NO3 oxidation of pure and mixed monoterpenes. *Atmos Chem Phys* **20**, 15513-15535, doi:10.5194/acp-20-15513-2020 (2020).
